# How Semantically Labeled Scent-Gender Associations Influence the Evaluations of Scent and Texture

**DOI:** 10.3389/fpsyg.2021.713329

**Published:** 2021-10-21

**Authors:** Sayo Iseki, Kosuke Motoki, Ryosuke Sakata, Shinji Kitagami

**Affiliations:** ^1^Department of Management, Chukyo University, Nagoya, Japan; ^2^Department of Food Science and Business, Miyagi University, Sendai, Japan; ^3^Department of Informatics and Sciences, Nagoya University, Nagoya, Japan; ^4^Department of Psychology, Graduate School of Informatics, Nagoya University, Nagoya, Japan

**Keywords:** haptics, olfaction, scent-gender associations, sensory marketing, multisensory experiences

## Abstract

Sensory evaluation can be influenced by semantic information such as gender descriptions. Gender categories are associated with tactile information (e.g., female = soft/smooth, while male = hard/rough). Feminine scents (e.g., floral) are typically perceived as soft and smooth. Thus, semantic labels of gender (feminine/masculine qualities) may influence congruent sensory evaluation (i.e., female = soft/smooth, male = hard/rough). This study examined how semantically labeled scent-gender associations influence the evaluation of scent and texture. Specifically, we examined whether “feminine” and “masculine” labels applied to neutral scents that have not been associated with gender influence scent and haptic evaluation. Participants sniffed a feminine-labeled or masculine-labeled scent embedded on soft and rough papers. They then evaluated the scent (e.g., gender perception) and texture (e.g., hedonic evaluation). The results demonstrated that participants who sniffed a feminine-labeled (vs. masculine-labeled) scent perceived it as more feminine. However, contrary to our expectations, gender labeling of scent did not influence haptic evaluation. These findings indicate that semantic labeling of scents (i.e., feminine/masculine) may alter the gender perception of a scent but not the tactile evaluation. Practical implications for (online) sensory marketing are discussed.

## Introduction

Many fabric softeners and hand creams are imbued with floral scents. This makes it important to investigate how these types of scents influence texture perception, which has been shown to be multisensory in olfaction and haptics (Spence and Bremner, 2011). People map features from one sensory modality onto features in other sensory modalities in a surprisingly consistent manner (Spence, 2011). This phenomenon is referred to as cross-modal correspondence (Spence, 2011). For example, previous research on multisensory perception has shown the existence of cross-modal interactions between tactile information and other sensory modalities, including light (e.g., Guest and Spence, 2003), sound (e.g., Guest et al., 2002; Uchida et al., 2021), and taste (e.g., Wang and Spence, 2018; Carvalho et al., 2020; Pramudya et al., 2020). Several studies have specifically shown that olfaction (smell) can also interact with tactile perception (e.g., Laird, 1932; Demattè et al., 2006; Luisa Demattè et al., 2007; Koijck et al., 2015). For example, fabrics containing lemon scents are perceived as softer than those containing animal scents (Demattè et al., 2006).

As for texture, tactile information is associated with gender categories. Specifically, textures provide metaphorical associations for representations of gender categories (e.g., Feingold, 1994; Blair et al., 2001; Gawronski and Bodenhausen, 2005), in which the male and female genders are typically associated with “tough” and “tender,” respectively (Blair et al., 2001; Gawronski and Bodenhausen, 2005). For example, Slepian et al. (2011) found that gender-ambiguous faces were more likely to be judged as female when grasping a soft ball as opposed to a hard ball. These findings suggest common gender-tactile associations, in which “female” is equated with “soft/smooth,” while “male” is equated with “hard/rough.”

The semantic label of femininity/masculinity on scents influences perception and cognition (e.g., Zellner et al., 2008; Speed and Majid, 2019; Spence, 2021). Zellner et al. (2008) demonstrated that femininity/masculinity labels on fine fragrances influenced color–odor correspondences, with participants being told that a fragrance was either feminine (for women) or masculine (for men). In Zellner et al. (2008), participants were told that the fragrance was either feminine (for women) or masculine (for men). When participants were told the fragrances were for females, they tended to judge the fragrances as feminine and think of lighter/feminine colors (e.g., pink; Taft, 1997). In contrast, when participants were told the fragrances were for male, they tended to consider the fragrances masculine and think of darker/masculine colors (e.g., blue; Taft, 1997). This suggests that semantic feminine/masculine labels on scents can alter their color associations.

In this study, we examine whether the effects of semantic feminine/masculine labels on scents (Zellner et al., 2008) could be conceptually replicated. Based on the evidence described above, we establish

Hypothesis I: A feminine labeled scent will be perceived as more feminine than a masculine-labeled scent.

We construct a second hypothesis following Krishna et al. (2010), who demonstrated that a semantic congruence between smell and touch results in a higher evaluation of the tactile quality of a given texture. Because smooth and rough papers are, respectively, perceived as feminine and masculine, there was a semantic congruence between smell and touch on smooth/rough paper soaked with feminine/masculine scents. When the smell is feminine, participants thus tend to rate the tactile quality of smooth paper more positively than when the smell is masculine, and vice versa. Furthermore, Adams and Doucé (2017) demonstrated that several scents associated with femininity (or masculinity) are strongly interrelated with the expectation of smooth (or rough) by using an assortment of 32 scents. Thus, we establish

Hypothesis II: Semantic congruence between gender labels and touch will result in a higher hedonic evaluation of haptics. Specifically, feminine-labeled scents will increase the tactile evaluation of smooth paper, while masculine-labeled scents will increase the tactile evaluation of rough paper.

With these concepts in mind, this study examines the role of semantic labeling of scents in haptic evaluations involving a stimulus, in this case paper. More specifically, we examine whether “feminine” and “masculine” labels influence haptic perceptions and hedonic evaluations of paper imbued with neutral scents.

## Methods

### Preliminary Study

We conducted a preliminary study to select neutral scents (neither associated with femininity nor masculinity) for use in our main study. We recruited a total of 46 university students (27 males, 19 females; mean age of 20.39 years, *SD* = 2.43). None of these participants answered that they were allergic to fragrances or scents. The study design was a one-factor, within-participants design.

The stimuli included five commercially available unisex perfumes with varying degrees of familiarity, as follows: (a) Calvin Klein CK One, (b) CLEAN Reserve Warm Cotton, (c) Cartier Essence d’Orange, (d) CLEAN Reserve Rain, and (e) HERMES Concentré d’Orange Verte. We soaked 0.3 ml of each perfume into individual pieces of cotton that were stored in resealable bags (Krishna et al., 2010).

Participants answered the following questionnaire for each stimulus. The questionnaire contained four items, each of which were rated on a 9-point Likert scale:

• Gender perception of scent (“1: extremely masculine” to “9: extremely feminine”)
• Preference (“1: extremely dislike” to “9: extremely like”)
• Pleasantness (“1: extremely unpleasant” to “9: extremely pleasant”)
• Familiarity (“1: extremely unfamiliar” to “9: extremely familiar”).

Participants also answered three post-test questions^1^ :

• “Do you think the scent you smelled in this survey was perfume?” (Yes, No, Neither)
• “Do you have an interest in perfume?” (“1: not interested at all” to “5: very interested”)
• “Do you usually wear perfume?” (every day, once a week, once a month, several times a year, not at all).

The survey was conducted on an individual basis, with each participant asked to turn the paper questionnaire pages according to the researcher’s instructions. In addition, in front of each participant was a small white cup half-full with coffee beans. Following the method implemented by Krishna et al. (2010), after opening a sealed bag containing a given stimulus (scented cotton), participants smelled the contained scent three times. Next, they were asked to answer the four abovementioned items (gender perception, preference, pleasantness, and familiarity). No information about scents were given to the participants. These procedures were repeated for each of the five stimuli. The order in which the stimuli were presented was counterbalanced between participants, although the order of the four items was fixed. Following Krishna et al. (2010), participants were asked to refresh their olfactory sense by smelling coffee beans in a paper cup prior to smelling any successive stimulus.

Means and standard deviations were calculated for the gender perception of the scents (Supplementary Table 1).^2^ For each of the five scents, one-sample *t*-tests (two-tailed) were performed with the gender perception of scents set as the dependent variable. Specifically, one-sample *t*-tests were conducted to determine whether the gender perception of the scents significantly differed from the midpoint (5). Here, the results showed that gender perceptions did not significantly differ from the midpoint for either Cartier Essence d’Orange (Cartier) or HERMES Concentré d’Orange Verte (HERMES), thus indicating that each were perceived as neutral scents (see Supplementary Table 1). Further, we conducted paired samples *t*-tests to investigate any differences between Cartier and HERMES in regard to preference, pleasantness, and familiarity. For preference, HERMES was rated higher than Cartier [Cartier: *M* = 5.54, *SE* = 0.19; HERMES: *M* = 6.02, *SE* = 0.19; *t*(45) = 2.08, *p* = 0.04, *dz* = 0.24]. For pleasantness, HERMES was also rated higher than Cartier [Cartier: *M* = 5.46, *SE* = 0.18; HERMES: *M* = 6.00, *SE* = 0.16; *t*(45) = 2.59, *p* = 0.01, *dz* = 0.30]. For familiarity, however, no difference was observed between Cartier and HERMES [tier: *M* = 4.98, *SE* = 0.23; HERMES: *M* = 4.96, *SE* = 0.26; *t*(45) = 0.07, *p* = 0.94, *dz* = 0.009]. Given these findings, we determined that HERMES was the best stimulus for use in the main study. The results of the three post-test questions are provided in the foot notes.^3^

### Main Study

#### Participants and Design

A two-factor mixed design was used for the main study (gender labeling for scent: feminine, masculine; between-participants factor) × (paper type: smooth, rough; within-participants factor). A total of 119 university students in Japan participated in the study. When asked, none of the participants stated that they were allergic to fragrances or scents. However, data from one participant were excluded due to missing values, meaning the final analyzed sample included 118 students (57 males, 61 females; mean age 18.90 years, *SD* = 0.65).

#### Materials

**FIGURE 1:**
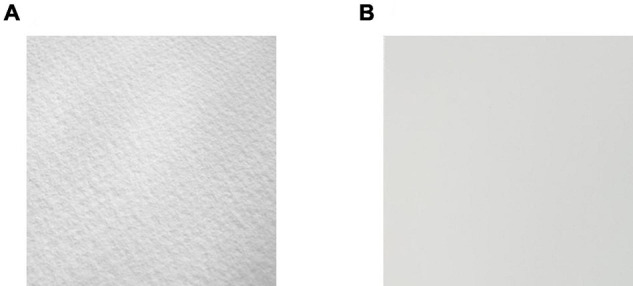
Rough and Smooth paper used as stimulus in the main study. **(A)** iPhoto S Morrow Coarse, size 100 × 148 mm, thickness 300 g/m^2^ (https://www.takeo.co.jp/en/). **(B)** IJ Livre Thick, size 100 × 148 mm, thickness 256 g/m^2^ (https://www.takeo.co.jp/en/).

The stimuli included smooth and rough postcard-sized papers, each of which were treated with 0.30 ml of HERMES perfume before being stored in sealable bags (Krishna et al., 2010). The papers were sold by TAKEO Co., Ltd. (rough paper: iPhoto S Morrow Coarse, size 100 × 148 mm, thickness 300 g/m^2^; smooth paper: IJ Livre Thick, size 100 × 148 mm, thickness 256 g/m^2^; see Figure 1).

#### Procedures

The experiment was conducted in groups as part of a class. All participants provided written informed consent prior to participation. All study procedures adhered to the ethical standards of relevant institutional committees on human experimentation and the Declaration of Helsinki.

Participants were given paper questionnaires and instructed to turn the pages when instructed to do so by the researcher. They were randomly assigned to either the feminine condition (59 participants: 28 males, 31 females) or masculine condition (59 participants: 29 males, 30 females). First, they were presented with a mouillette (strip of paper) soaked in 0.3 ml of HERMES perfume, which had previously been stored in a sealed bag (Krishna et al., 2010). After smelling the scent, they were asked to read statements depending on their assigned condition. The feminine condition was as follows: “The mouillette you have received is scented with a perfume sold by a women’s brand.” On the other hand, the masculine condition was as follows: “The mouillette you have received is scented with a perfume sold by a men’s brand.” Regardless of the condition, all were asked to rate their impressions of the scent, including their gender perception (“1: extremely masculine” to “9: extremely feminine”) and preference (“1: extremely dislike” to “9: extremely like”).

**FIGURE 2:**
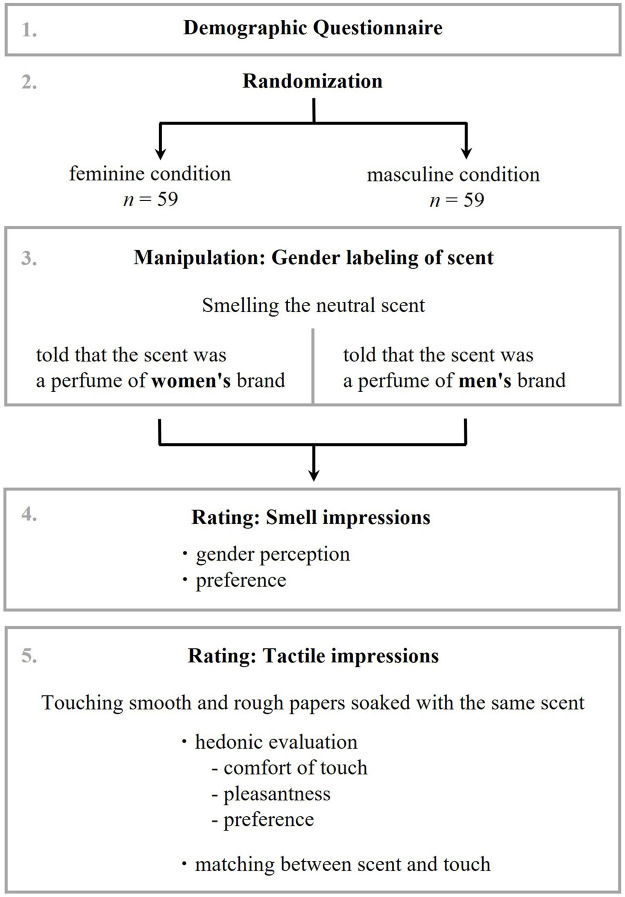
Flow chart of experimental procedure.

Participants were then presented with the abovementioned stimuli (smooth and rough papers) soaked with the same scent and asked to provide hedonic evaluation of haptics, including comfort of touch (“1: extremely bad” to “9: extremely good”), pleasantness (“1: extremely unpleasant” to “9: extremely pleasant”), and preference (“1: extremely dislike” to “9: extremely like”). Participants rated their hedonic evaluation of the haptics described above by touching the stimulus for about a minute. These procedures were repeated for each of the two types of stimuli (smooth and rough paper). Because only one fragrance was used in this study, there was no need to divide the experiment into multiple sessions to ventilate the remaining scents in the room. The order in which the stimuli were presented was counterbalanced between participants, although the order of the items remained fixed. To confirm the level of semantic congruence between scent and touch, participants were also asked to rate whether they thought the scent and touch matched for each stimuli (“1: not matched at all” to “9: very well matched”). The experimental procedure of this main study is depicted in Figure 2.

## Results

### Effects of Gender Labeling on Gender Perception of Scent

**FIGURE 3:**
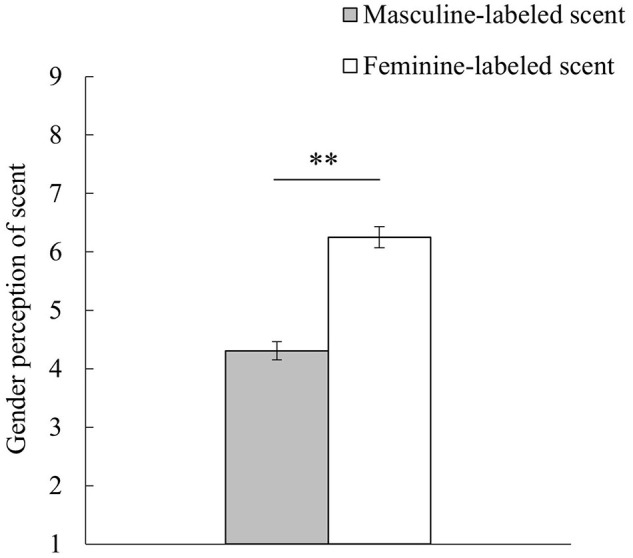
Effects of gender labeling on gender perception of scent. “1: extremely masculine” to “9: extremely feminine”; error bars indicate standard errors. ^∗∗^*p* < 0.01.

We conducted an unpaired *t*-test with gender labeling of scent (feminine or masculine) set as the independent variable, while gender perception of scent was set as the dependent variable. The results are shown in Figure 3. The feminine perception was significantly higher in the feminine condition than in the masculine condition [*t*(116) = 8.19, *p* < 0.001, *d* = 1.50]. We then conducted an additional *t*-test with the dependent variable set as preference but found no differences between the feminine (*M* = 5.19, *SE* = 0.18) and masculine (*M* = 5.48, *SE* = 0.20) [*t*(116) = 1.06, *p* = 0.29, *d* = 0.19] conditions in this regard. These findings show that participants in the feminine condition judged the scent as more feminine than those in the masculine condition, but this did not affect preference. This suggests that gender labeling for a neutral scent could create a gender-congruent semantic association.

### Semantic Congruence Between Scent and Touch

We conducted an ANOVA with gender labeling (feminine and masculine) and paper type (smooth and rough) set as independent variables, while the degree of match between scent and touch was set as the dependent variable. The main effect of gender labeling was not significant [*F*_(__1, 116)_ = 0.002, *p* = 0.96, η_*p*_^2^ = 0.00002], but the main effect of paper type was significant [*F*_(__1, 116)_ = 4.48, *p* = 0.04, η_*p*_^2^ = 0.04]. Here, rough paper (*M* = 5.44, *SE* = 0.15) was rated as more highly matched than smooth paper (*M* = 4.91, *SE* = 0.15), and no interactions were observed [*F*_(__1, 116)_ = 0.71, *p* = 0.40, η_*p*_^2^ = 0.01]. Based on these results, there was neither matching between rough paper and masculine scent nor between smooth paper and feminine scent.

### Effects of Gender Labeling of Scent on Hedonic Evaluation of Haptics

**FIGURE 4:**
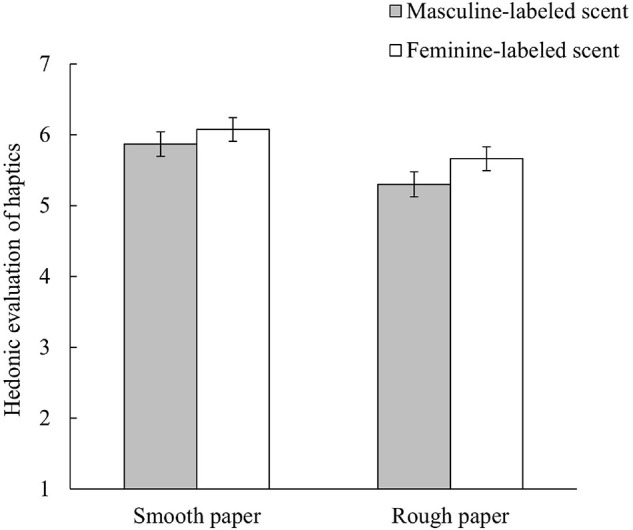
Effects of gender labeling of scent on hedonic evaluation of haptics. Error bars indicate standard errors.

The ratings of comfort of touch, pleasantness, and preference were averaged to calculate the hedonic evaluation of haptics (Cronbach’s α = 0.91).^4^ We conducted an analysis of variance (ANOVA) with gender labeling of scent (feminine or masculine) and paper type (smooth or rough) set as the independent variables, while hedonic evaluation of haptics was set as the dependent variable. The results are shown in Figure 4. The main effect of paper type was significant [*F*_(__1, 116)_ = 8.05, *p* = 0.01, η*_*p*_*^2^ = 0.07]. However, neither the main effect of gender labeling of scent [*F*_(__1, 116)_ = 2.80, *p* = 0.10, η*_*p*_*^2^ = 0.02] or interaction [*F*_(__1, 116)_ = 0.21, *p* = 0.65, η*_*p*_*^2^ = 0.002] were significant. These results did not support Hypothesis II.

## Discussion

This study aimed to examine how semantically labeled scent-gender associations influence the evaluation of scent and texture. Specifically, we examined whether “feminine” and “masculine” labels applied to neutral scents influenced scent perception and haptic evaluation of soft and rough paper. First, we examined whether semantic feminine/masculine labels on scents influence congruent gender perceptions of scent (Hypothesis I). The results demonstrated that the feminine labeled scent was perceived as more feminine than the masculine-labeled scent. Thus, Hypothesis I was supported. Second, we examined whether semantic congruence between gender labels and touch would result in a higher hedonic evaluation of haptics (Hypothesis II). The results revealed neither a semantic congruence between scent and touch nor congruence effects on hedonic evaluation of haptics. That is, feminine-labeled (masculine-labeled) scents were not matched with soft (rough) paper. Moreover, expected congruency between gender labels and touch (i.e., feminine-label and soft paper, masculine-label and hard paper) did not result in higher hedonic evaluation of haptics than incongruent ones (i.e., masculine -label and soft paper, feminine-label and rough paper). Therefore, Hypothesis II was not supported. Together, our findings reveal how semantically labeled scent-gender associations influence the evaluations of scent and texture.

The findings conceptually replicated Zellner et al. (2008) in a different context. Zellner et al. (2008) show that participants tend to judge fragrances as feminine (or masculine) when participants are told that the fragrances are for women (or for men). Our procedures employed a context associated with consumer behavior. Specifically, in our study, when the scent was presented, participants were told “This is a perfume sold by a women’s brand.” In contrast, in Zellner et al. (2008), participants were only told “This scent is for women.” Furthermore, the participants in our study were mostly Japanese, whereas those in Zellner et al. (2008) were mostly Caucasian from a university in America (e.g., in Experiment 4, the participants were 71% Caucasian, 9% African-American, 13% Hispanic, and 7% other). Additionally, our findings were obtained using different odor stimuli from Zellner et al. (2008). Our study used Hermes Concentré d’Orange Verte, while Zellner et al. (2008) employed Calvin Klein CKOne and CKBe as neutral scents. Together, although three points (i.e., context, culture, and scent) were different from Zellner et al. (2008), our findings conceptually replicated the semantically labeled scent-gender associations.

Our results indicate that odor perception is cognitively modulated. Indeed, previous research has shown that scent labels such as “cheddar cheese” and “body odor” can alter evaluations (De Araujo et al., 2005). Relevant to this study, Zellner et al. (2008) found that scent labeling (e.g., “feminine” or “masculine”) altered related gender perceptions. That is, a feminine-labeled scent induced more feminine perceptions, while a masculine-labeled scent induced more masculine perceptions. Our findings add to the existing body of evidence pertaining to the role of cognitive modulations in odor perception, particularly revealing that gender-labeled scents induce corresponding gender perceptions.

Our findings can provide practical implications for (online) sensory marketing. Our results indicate that semantic labeling for neutral scents that are not associated with feminine/masculine may alter gender perception. For neutral scents, marketers need to carefully use words related to gender in various contexts (e.g., product descriptions, advertisements). On the other hand, gender labeling should be proactively applied when marketing a neutral scent to women or men. Such labeling may be especially important in online marketing environments, where consumers cannot try scents to compare them with other scents before purchasing.

Marketers should be cautious about the branding of neutral scents (e.g., orange, strawberry, jasmine, sandalwood, and lavender; Adams and Doucé, 2017) using gender labeling. Such neutral scents can be used in products (e.g., fabric softeners, hand creams) whose textural attributes (e.g., softness, smoothness) have a significant role in consumer evaluations. When these neutral scents are sold by a women’s (or men’s) brand, the branding may not influence tactile evaluations.

This study also had some limitations. First, only one scent was used in our main study. As such, the generalizability of the results is not validated. In our preliminary study, Cartier Essence d’Orange was shown to be neutral (i.e., neither associated with femininity nor masculinity). To increase the generalizability of the results, further study using a different scent (e.g., Cartier Essence d’Orange) in needed. Second, we did not use a control condition (i.e., no labeling of gender categories). Our procedure was similar to Experiment 4 in Zellner et al. (2008), which included only two conditions (i.e., for women and for men) and no control condition. Nevertheless, future research needs to rigorously investigate the influence of gender labeling on gender perception of scent by using a control condition. Third, it is also important to carefully identify participants with allergies to scents and smell disorders using the Olfactory Assessment Test (Nordin et al., 2003). Moreover, although there was a nearly equal ratio of male to female participants in the main study (57 males, 61 females), a gender balanced sample was not adopted in the preliminary study (27 males, 19 females). Furthermore, demographic variables such as age and culture were restricted to a homogeneous population. That is, almost all participants were university students (*M* = 18.90 years, *SD* = 0.65) of Japanese nationality, which diminishes the generalizability of our results. Finally, we did not consider cultural dependencies (e.g., culturally different perceptions of everyday odors; Ayabe-Kanamura et al., 1998). This makes it important to conduct additional studies among different populations and in different areas.

## Data Availability Statement

The raw data supporting the conclusions of this article will be made available by the authors, without undue reservation.

## Ethics Statement

The studies involving human participants were reviewed and approved by the Ethics Committee at Nagoya University. The patients/participants provided their written informed consent to participate in this study.

## Author Contributions

SI: conceptualization, formal analysis, and funding acquisition. SI, KM, and RS: methodology. SI and KM: writing the original draft. SI, RS, and SK: investigation and data curation. SK: resources and supervision. SI, KM, and SK: writing, reviewing, and editing. All authors contributed to the manuscript and approved of the submitted version.

## Conflict of Interest

The authors declare that the research was conducted in the absence of any commercial or financial relationships that could be construed as a potential conflict of interest.

## Publisher’s Note

All claims expressed in this article are solely those of the authors and do not necessarily represent those of their affiliated organizations, or those of the publisher, the editors and the reviewers. Any product that may be evaluated in this article, or claim that may be made by its manufacturer, is not guaranteed or endorsed by the publisher.

## Footnotes

1 The first question was asked to check whether they had noticed that the presented scents were derived from perfumes. The second and third questions were asked to investigate the individual characteristics of the participants in terms of their attitudes and daily behaviors toward perfumes.

2 For each of the five scents, unpaired *t*-tests (two-tailed) were performed with the gender of the participants set as the independent variable, while gender perception of scent was set as the dependent variable. The results showed that gender perceptions of scent did not significantly differ between male and female participants for each of the five scents (*p* > 0.10).

3 Seventy percent of the participants noticed that the presented scents were perfumes. The participants’ interest in perfumes was moderate (*M* = 2.76, *SD* = 1.15), and 54% of participants reported that they usually did not wear perfume at all.

4 As demonstrated in Supplementary Table 2, three dimensions (i.e., comfort of touch, pleasantness, and preference) were significantly correlated with each other. In addition, we performed a principal component analysis with oblimin rotation on the three items used to measure hedonic evaluation of haptics. The solution produced one factor, explaining 84.76% of variance (factor loadings: comfort of touch: 0.89, pleasantness: 93, preference: 94).
