# Supplementary material for: How Semantically Labeled Scent-Gender Associations Influence the Evaluations of Scent and Texture
Source: Front Psychol. 2021 Oct 21;12:713329. doi: 10.3389/fpsyg.2021.713329 (PMC8566334; doi:10.3389/fpsyg.2021.713329)
Supplement: Supplementary file 1 [file Table_1.docx]

Supplementary Material

Supplementary Table 1. Descriptive statistics and *t*-test results for gender perception of scent

| v | *M* | *SD* | *t* (45) | *p*-value |
| --- | --- | --- | --- | --- |
| Calvin Klein CK One | 5.65 | 1.94 | 2.29 | .03 |
| CLEAN Reserve Warm Cotton | 6.00 | 1.66 | 4.09 | < .001 |
| Cartier Essence d’Orange | 5.37 | 1.53 | 1.64 | .11 |
| CLEAN Reserve Rain | 5.87 | 1.64 | 3.59 | < .001 |
| HERMES Concentré d’Orange Verte | 4.85 | 1.48 | 0.70 | .49 |

Note. Means and standard deviations were calculated for gender perception of scent. For each of the five listed scents, one-sample *t*-tests (two-tailed) were then performed with gender perception of scent as the dependent variable, thus providing a way to determine whether the gender perception of scent significantly differed from the midpoint (5).

# Supplementary Table 2. Correlation Coefficients Between Three Dimensions of Hedonic Evaluation of Haptics

|  | *M* | *SD* | 1 |  | 2 |  | 3 |  |
| --- | --- | --- | --- | --- | --- | --- | --- | --- |
| 1. Comfort of touch | 5.78 | 1.06 | ─ |  |  |  |  |  |
| 2. Pleasantness | 5.72 | 0.94 | .73 | ^**^ | ─ |  |  |  |
| 3. Preference | 5.69 | 1.03 | .74 | ^**^ | .84 | ^**^ | ─ |  |

^**^*p* < .01
